# Integrating the dysregulated inflammasome-based molecular functionome in the malignant transformation of endometriosis-associated ovarian carcinoma

**DOI:** 10.18632/oncotarget.23364

**Published:** 2017-12-18

**Authors:** Chia-Ming Chang, Mong-Lien Wang, Kai-Hsi Lu, Yi-Ping Yang, Chi-Mou Juang, Peng-Hui Wang, Ren-Jun Hsu, Mu-Hsien Yu, Cheng-Chang Chang

**Affiliations:** ^1^ School of Medicine, National Yang-Ming University, Taipei, Taiwan; ^2^ Department of Obstetrics and Gynecology, Taipei Veterans General Hospital, Taipei, Taiwan; ^3^ Department of Medical Research, Taipei Veterans General Hospital, Taipei, Taiwan; ^4^ Department of Medical Research and Education, Cheng-Hsin Hospital, Taipei, Taiwan; ^5^ Department of Medical Research, China Medical University Hospital, Taichung, Taiwan; ^6^ Graduate Institute of Life Sciences, National Defense Medical Center, Taipei, Taiwan; ^7^ Biobank Management Center of Tri-Service General Hospital, National Defense Medical Center, Taipei, Taiwan; ^8^ Department of Obstetrics and Gynecology, Tri-Service General Hospital, National Defense Medical Center, Taipei, Taiwan

**Keywords:** endometriosis, ovarian carcinoma, inflammasome, gene-set integrative analysis, gene expression microarray

## Abstract

The coexistence of endometriosis (ES) with ovarian clear cell carcinoma (CCC) or endometrioid carcinoma (EC) suggested that malignant transformation of ES leads to endometriosis associated ovarian carcinoma (EAOC). However, there is still lack of an integrating data analysis of the accumulated experimental data to provide the evidence supporting the hypothesis of EAOC transformation. Herein we used a function-based analytic model with the publicly available microarray datasets to investigate the expression profiling between ES, CCC, and EC. We analyzed the functional regularity pattern of the three type of samples and hierarchically clustered the gene sets to identify key mechanisms regulating the malignant transformation of EAOC. We identified a list of 18 genes (NLRP3, AIM2, PYCARD, NAIP, Caspase-4, Caspase-7, Caspase-8, TLR1, TLR7, TOLLIP, NFKBIA, TNF, TNFAIP3, INFGR2, P2RX7, IL-1B, IL1RL1, IL-18) closely related to inflammasome complex, indicating an important role of inflammation/immunity in EAOC transformation. We next explore the association between these target genes and patient survival using Gene Expression Omnibus (GEO), and found significant correlation between the expression levels of the target genes and the progression-free survival. Interestingly, high expression levels of AIM2 and NLRP3, initiating proteins of inflammasomes, were significantly correlated with poor progression-free survival. Immunohistochemistry staining confirmed a correlation between high AIM2 and high Ki-67 in clinical EAOC samples, supporting its role in disease progression. Collectively, we established a bioinformatic platform of gene-set integrative molecular functionome to dissect the pathogenic pathways of EAOC, and demonstrated a key role of dysregulated inflammasome in modulating the malignant transformation of EAOC.

## INTRODUCTION

Epithelial ovarian carcinomas (EOCs) are composed of a group of heterogeneous subtypes classified by their histology and the degree of epithelial proliferation and invasion [1]. Among these subtypes, clear cells carcinoma (CCC) and endometrioid carcinoma (EC) share many similarities in their tumor behavior, clinical features, and pathology. Endometriosis (ES) is found in 15%-20% of CCC and EC, and is associated with 2-3 fold increase of EOC incidence [2] [3]. The atypical ES, characterized by large nuclei and increased nuclear-cytoplasmic ratio, composes 8% of ES [4] and is found in 36% and 23% in CCC and EC, respectively [5]. Atypical ES was shown direct continuity with CCC and EC and is considered to be a precancerous transformation process of CCC and EC [6]. These clinical observations indicate a close relationship between ES and CCC/EC, and support the hypothesis of endometriosis associated ovarian carcinoma (EAOC). Recent genomic studies have greatly increased our understanding of the molecular landscape of EOC [7] [8]. However, the molecular pathogenesis involving in the malignant transformation from ES to EAOC is still unclear.

The Sampson’s theory of retrograde menstruation is the most widely accepted theory on the pathogenesis of ES [9]. However, there exists a paradox: although retrograde menstruation is widely encountered among reproductive women, the incidence of ES is relatively uncommon compared with the manifestation of retrograde menstruation experienced by most of the women in the same group [10]. One hypothesis is that in comparison to women without ES, the women that develop ES have a defective immune system unable to recognize the endometrial fragments within the pelvic cavity. Inflammatory responses play key roles at different stages of tumor development, including initiation, promotion, malignant conversion, invasion, and metastasis. Inflammation also disturbs immune surveillance and tumor responses to therapy. Immune cells that infiltrate tumors involve in a dynamic crosstalk with cancer cells and some of the molecular consequences that mediate this dialog have been identified [11].

The Gene Ontology (GO) [2] is the primary tool to annotate the gene products and enable the functional interpretation of the genomic data. It defines relatively comprehensive human functionome like biological processes, molecular functions and cellular components. This gene set regularity (GSR) model has been successfully utilized to demonstrate the dualistic model of ovarian carcinogenesis [12], and to quantify the function deterioration of the FIGO staging I to IV for serous ovarian carcinoma [13]. In this study, we investigated the dysregulated functions involving in the malignant transformation from ES to EAOC with GSR model by analyzing the functionomes consisted of 5917 GO defined functions of ES, CCC and EC with the DNA microarray datasets downloaded from the publicly available database. The results demonstrated that the immune/inflammation related functions were crucial elements involving in the transformation of EAOC. Among these dysregulated immune/inflammation related functions, the inflammasome complex (G0:0061702) is noticeable because it is postulated to become activated during malignant transformation of tumorigenesis and plays diverse roles in cancer promotion [14]. To study the role of inflammasome complex in the malignant transformation from ES to EACO, we explored the expressions of the inflammasome related genes by carrying out an integrative analysis with the same DNA microarray expression datasets. The results revealed several inflammasome complex and inflammasome-related genes (NLR Family Pyrin Domain Containing 3 (NLRP3), Absent In Melanoma 2 (AIM2), PYD And CARD Domain Containing (PYCARD), NLR Family Apoptosis Inhibitory Protein (NAIP), Tumor Necrosis Factor (TNF), Toll Like Receptor 1 (TLR1), Toll Like Receptor 7 (TLR7), Toll Interacting Protein (TOLLIP), and NFKB Inhibitor Alpha (NFKBIA)) differentially expressed in ES, CCC and EC, and significantly correlating with poor progression-free survival. The expression levels of these identified genes were confirmed by immunohistochemistrical staining in ES, CCC and EC specimens. These findings are vital to clarify the role of inflammasome in EAOC carcinogenesis.

## RESULTS

### Workflow of the study

**Figure 1:**
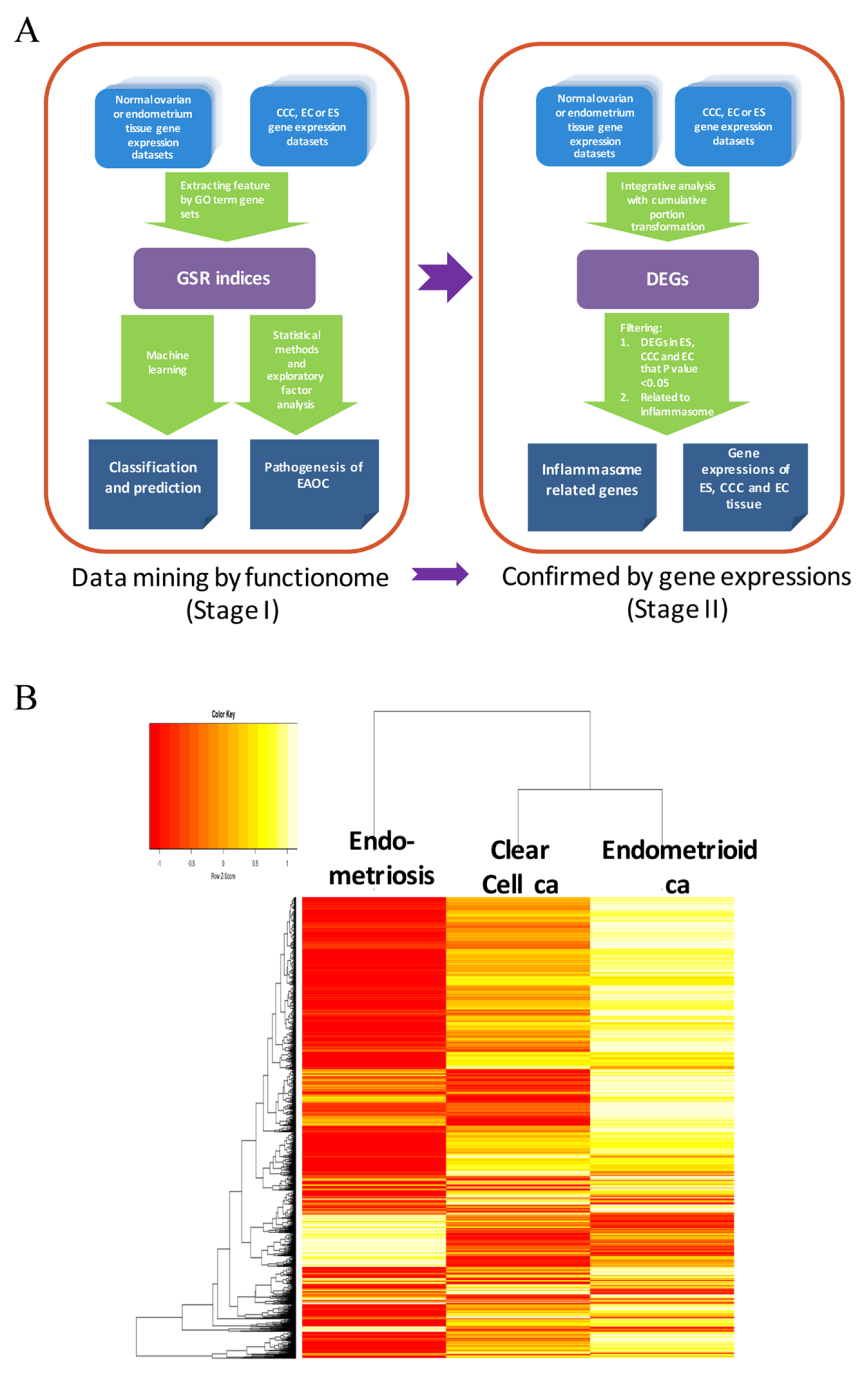
Work flow of the two-stage strategy to discover gene signatures for EAOC **(A)** Workflow of the gene set regularity model. The gene set regularity (GSR) index was computed by converting the gene expression ordering of gene elements in a gene set through the Gene Ontology (GO) term or canonical pathway databases. The informativeness of the GSR index was assessed by the accuracy of recognition, classification, and prediction by machine learning using binary or multiclass classifications. Functionome analyses were carried out to investigate the pathogenesis of endometriosis (ES), clear cell carcinoma (CCC), endometrioid ca (EC) and endometriosis-associated ovarian carcinoma (EAOC) by statistical methods, hierarchical clustering, and exploratory factor analysis. **(B)** Heatmaps and dendrogram for the three diseases. The dendrogram (left side of the heatmap) showed the relationship of the three diseases. When displayed on the heatmap, each of the three diseases computed through either the GO term gene sets showed a distinct pattern. However, the patterns were more similar between CCC and EC.

We utilize a two-stage strategy to discover the gene signatures involving in the transformation of EAOC, that is, starting with investigating the functionomes of ES, CCC and EC with the GSR model, and then followed by extracting the differentially expressed genes (DEGs) involving in these deregulated functions with integrative analysis. During the first stage, the GSR model was applied to find out the deregulated function related to the malignant transformation, it consisted with 4 steps as displayed on the left side of Figure 1A. First, extraction of expression profiles of gene set elements. The gene expression profiles for a given gene set were extracted from the publicly available microarray datasets according to the gene elements defined by each gene set. Second, computing GSR indices. The extracted gene expression profiles were converted to quantified functions based on the gene expression orderings of the gene elements in each gene set defined by the 5917 GO terms. This quantified function, i.e. the GSR index, is a measurement of the expression regularity of the genes in that gene set. The quantified functions range from 0 to 1; 1 represented unchanged regularity in a given gene set between the case and the most common gene expression orderings in the normal controls, while 0 represented the most chaotic state of the gene set regularity. Third, validating the functional regularity patterns. The informativeness of the functionome consisted of the 5917 GSR indices is evaluated by the accuracies of classification and prediction by the machine learning. Finally, investigation of EAOC pathogenesis. In this step, the key deregulated functions involving in the malignant transformation of ES to CCC or EC are investigated by a secession of analytic procedures. During the second stage (right side of Figure 1A), an integrative analysis of DNA microarray was applied to detect the differentially expressed genes. Then the principle genes involving in the malignant transformation of EAOC were filtered from those genes related to the deregulated functions detected by the first stage of analysis.

The microarray gene expression profiles of ES, CCC, EC and the normal control samples were downloaded from the GEO database, including 107 ES, 156 normal endometrium controls, 85 CCC, 90 EC, and 136 normal ovarian tissue control samples (Table 1). These samples data were collected from 39 datasets containing 7 different DNA microarray platforms without missing data. The detailed sample information, including the DNA microarray platforms, dataset series, and accession number, were listed in Supplementary Table 1. Because different genes utilized in different microarray platforms, a total of 5905 common gene sets were utilized finally for the GSR model in this study. Table 1 displays the sample number, mean and standard deviation (SD) of the GSR indices for the three diseases and the normal tissue controls. The means of GSR indices for the three diseases were significantly lower than the controls, indicating that the functions are generally deteriorated in the ES, CCC or EC when comparing with the normal control group. The informativeness of the GSR indices was evaluated by the accuracies of classification and prediction for the functional regularity patterns of the three diseases. Supervised classification was performed by support vector machine (SVM) and the performance was assessed by the accuracies of the binary and multiclass classification of the GSR matrices computed from the total samples through 5905 GO term gene sets. The performance was tested by five-fold cross validation. The results showed up to 100% accuracies of binary classification (case vs control). The area under curves (AUCs) ranged from 0.98 to 1 (Table 2). The accuracies of multiclass classification among the three diseases and the normal control group were 98.68%. The high accuracies indicated that the GSR indices could provide sufficient information for the machine learning to recognize and undergo adequate recognition and classification. It also revealed distinct functional regularity patterns of ES, CCC and EC, which can be applied to the molecular classification among ES, CCC and EC. Unsupervised classification by the hierarchical clustering was performed to uncover the relationship between the three diseases (Figure 1B). The clustering data revealed a relatively close relationship between CCC and EC, and the detailed dendrogram of the GO terms were shown in Supplementary Figure 1. The heatmap (Figure 1B) also showed similar patterns between CCC and EC. There were many overlapped deregulated molecular functions and biological processes between CCC and EC, indicating a close etiology of these two types of cancer.

**Table 1:** Sample number and mean of the gene set regularity indexes for each group

Disease	Case	Control	Case Mean (SD)	Control Mean (SD)	P value
**ES**	107	156	0.6299(0.0832)	0.6715(0.0825)	<2.2x10^-16^
**CCC**	85	136	0.6304(0.1034)	0.6532(0.1120)	<2.2x10^-16^
**EC**	90	136	0.6466(0.0.1051)	0.6539(0.1116)	<2.2x10^-16^

**Table 2:** Accuracies of the binary and multiclass classification and prediction by machine learning

Gene set	Classification	Group	Sensitivity(SD)	Specificity(SD)	Accuracy(SD)	AUC
**GO term**	**Binary**	**ES**	1.0000(0.0000)	1.0000(0.0000)	1.0000(0.0000)	1.0000
		**CCC**	1.0000(0.0000)	1.0000(0.0000)	1.0000(0.0000)	1.0000
		**EC**	0.9597(0.0303)	0.9965(0.0109)	0.9800(0.0163)	0.9768
	**Multiclass**	**ES-CCC-EC- control**	NA	NA	0.9868(0.0046)	NA

### Discovering the deregulated functions involving in the malignant transformation of EAOC by mining the DNA microarray gene expression data

**Figure 2:**
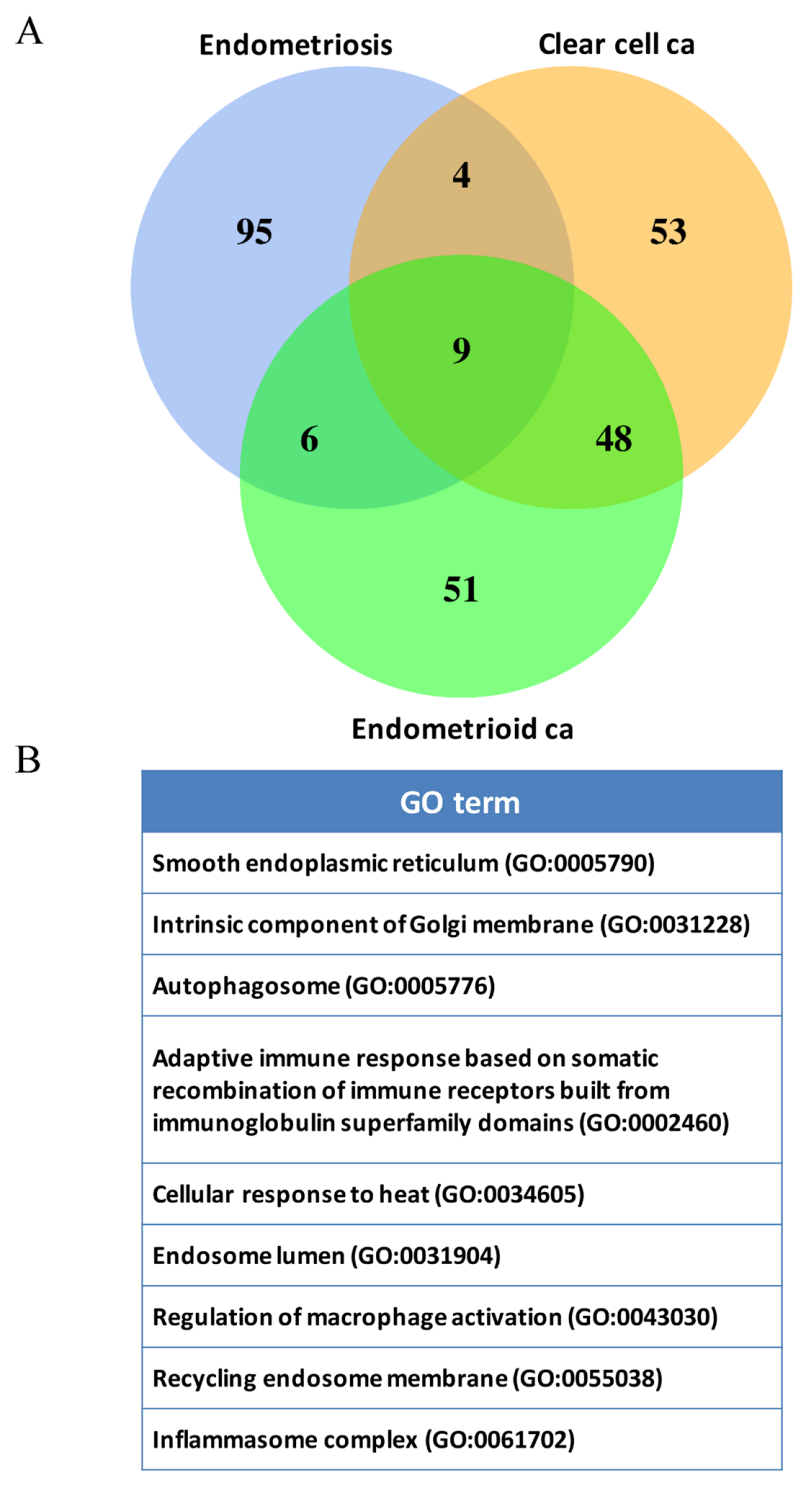
DNA microarray gene expression data mining of deregulated functions involving in the malignant transformation of EAOC **(A)** Venn diagram of the deregulated GO term elements from exploratory factor analysis for the three diseases. The figure showed the results of the three diseases with the total factor elements from each of the disease. Their relationship was displayed on the Venn diagram to show the gene set numbers of all possible logical relations among the three diseases. The 9 commonly deregulated GO terms among ES, CCC and EC were listed on the right side of the figure. **(B)** The nine commonly deregulated GO terms among the ES, CCC, and EC, including ‘inflammasome complex’ was shown.

We used the set operations to identify commonly deregulated functions from the top 1000 significantly deregulated GO terms among ES, CCC and EC. There were 65 deregulated functions in common (Supplementary Table 2), revealing the possible etiology of malignant transformation of EAOC. Among the 65 deregulated functions, up to 16.9% (11/65) deregulated functions were relating to inflammation/immune, showing the important roles of inflammation/immune playing on the malignant transformation of EAOC. We then focused on the immune/inflammation related functions and extracted them from the functionomes of ES, CCC and EC using the following keys: ‘immune system process’ (GO:0002376), ‘response to stress’ (GO:0006950), ‘cytoplasmic part’ (GO:0044444), and ‘cytokine production’ (GO:0001816) to collect all of their offspring. Table 3 displayed the 114 most significantly deregulated immune/inflammation related GO terms in the three diseases. These immune/inflammation related GO terms were predominately associated with deregulated cytokines production, signaling pathways and activation of immune cells. We carried out the set operations with the 114 GO terms to discover the coexisting immune/inflammation related GO terms among ES, CCC and EC, and displayed the results on the Venn diagram in Figure 2A. The detailed information of the 114 genes were available in Supplementary Table 3. The CCC and EC shared the most number of overlapping deregulated GO terms, accounting for 50% (57/114) of the coexisting deregulated GO terms, indicating the similar immune pathogenesis between these two cancers. There were 9 commonly deregulated GO terms among the ES, CCC and EC as shown on the Figure 2B.

**Table 3:** The 114 most deregulated immune/inflammation related Gene Ontology terms for the three diseases ranked by the P values

	Endometriosis	Clear cell carcinoma	Endometrioid carcinoma
**1**	Golgi cisterna GO:0031985	Negative regulation of antigen receptor mediated signaling pathway GO:0050858	Regulation of B cell receptor signaling pathway GO:0050855
**2**	Golgi stack GO:0005795	Endoplasmic reticulum quality control compartment GO:0044322	Wound healing spreading of epidermal cells GO:0035313
**3**	Positive regulation of interleukin 2 biosynthetic process GO:0045086	Regulation of B cell receptor signaling pathway GO:0050855	Regulation of Toll like receptor 4 signaling pathway GO:0034143
**4**	Interferon gamma mediated signaling pathway GO:0060333	Regulation of natural killer cell activation GO:0032814	Regulation of oxidative stress induced neuron death GO:1903203
**5**	Response to interferon gamma GO:0034341	Mast cell granule GO:0042629	Lamellar body GO:0042599
**6**	Cellular response to interferon gamma GO:0071346	Positive regulation of endoplasmic reticulum unfolded protein response GO:1900103	Smooth endoplasmic reticulum GO:0005790
**7**	Adaptive immune response based on somatic recombination of immune receptors built from immunoglobulin superfamily domains GO:0002460	Platelet alpha granule lumen GO:0031093	Negative regulation of antigen receptor mediated signaling pathway GO:0050858
**8**	Positive regulation of interleukin 8 production GO:0032757	Lysosomal lumen GO:0043202	T cell differentiation involved in immune response GO:0002292
**9**	Intrinsic component of endoplasmic reticulum membrane GO:0031227	Smooth endoplasmic reticulum GO:0005790	Angiogenesis involved in wound healing GO:0060055
**10**	Regulation of interleukin 8 production GO:0032677	Regulation of Toll like receptor 4 signaling pathway GO:0034143	Regulation of IRE1 mediated unfolded protein response GO:1903894
**11**	Lymphocyte mediated immunity GO:0002449	Platelet alpha granule GO:0031091	Endoplasmic reticulum quality control compartment GO:0044322
**12**	Golgi cisterna membrane GO:0032580	Vacuolar lumen GO:0005775	Methylosome GO:0034709
**13**	Recycling endosome GO:0055037	Positive regulation of macrophage activation GO:0043032	Interferon gamma production GO:0032609
**14**	Regulation of T helper cell differentiation GO:0045622	Recycling endosome GO:0055037	Regulation of response to interferon gamma GO:0060330
**15**	Clathrin coated endocytic vesicle GO:0045334	Secretory granule lumen GO:0034774	Intrinsic component of Golgi membrane GO:0031228
**16**	Lytic vacuole membrane GO:0098852	Regulation of antigen receptor mediated signaling pathway GO:0050854	Positive regulation of endoplasmic reticulum unfolded protein response GO:1900103
**17**	Regulation of cellular response to heat GO:1900034	Humoral immune response GO:0006959	Autophagosome GO:0005776
**18**	Late endosome GO:0005770	ER to Golgi transport vesicle GO:0030134	Positive regulation of transcription from RNA polymerase II promoter in response to stress GO:0036003
**19**	Stress activated protein kinase signaling cascade GO:0031098	Gamma tubulin complex GO:0000930	Regulation of endoplasmic reticulum unfolded protein response GO:1900101
**20**	Autophagosome GO:0005776	IRE1 mediated unfolded protein response GO:0036498	Axon regeneration GO:0031103
**21**	Recycling endosome membrane GO:0055038	Endoplasmic reticulum Golgi intermediate compartment GO:0005793	Regulation of T cell chemotaxis GO:0010819
**22**	Leukocyte mediated immunity GO:0002443	Cellular response to topologically incorrect protein GO:0035967	Humoral immune response GO:0006959
**23**	Smooth endoplasmic reticulum GO:0005790	Mast cell mediated immunity GO:0002448	Trans Golgi network membrane GO:0032588
**24**	Positive regulation of t helper cell differentiation GO:0045624	Myeloid leukocyte mediated immunity GO:0002444	Trans Golgi network transport vesicle membrane GO:0012510
**25**	Humoral immune response mediated by circulating immunoglobulin GO:0002455	Mast cell activation GO:0045576	Mature B cell differentiation GO:0002335
**26**	JNK cascade GO:0007254	Intrinsic component of mitochondrial outer membrane GO:0031306	Vesicle coat GO:0030120
**27**	Positive regulation of cd4 positive alpha beta T cell activation GO:2000516	Lamellar body GO:0042599	Complement activation GO:0006956
**28**	Endosomal part GO:0044440	Spleen development GO:0048536	Neutrophil mediated immunity GO:0002446
**29**	Response to heat GO:0009408	Regulation of endoplasmic reticulum unfolded protein response GO:1900101	Microbody lumen GO:0031907
**30**	Vacuolar membrane GO:0005774	Regulation of IRE1 mediated unfolded protein response GO:1903894	Adaptive immune response based on somatic recombination of immune receptors built from immunoglobulin superfamily domains GO:0002460
**31**	Cellular response to heat GO:0034605	Autophagosome GO:0005776	Clathrin coat of trans Golgi network vesicle GO:0030130
**32**	Regulation of cd4 positive alpha beta T cell activation GO:2000514	Negative regulation of T cell proliferation GO:0042130	Humoral immune response mediated by circulating immunoglobulin GO:0002455
**33**	Endosome GO:0005768	Toll like receptor 4 signaling pathway GO:0034142	Cd4 positive alpha beta T cell activation GO:0035710
**34**	Vacuolar part GO:0044437	ER associated ubiquitin dependent protein catabolic process GO:0030433	Trans Golgi network transport vesicle GO:0030140
**35**	Antigen processing and presentation GO:0019882	Positive regulation of transcription from RNA polymerase ii promoter in response to stress GO:0036003	Cellular response to heat GO:0034605
**36**	Endosome lumen GO:0031904	Negative regulation of T cell differentiation GO:0045581	Regulation of acute inflammatory response GO:0002673
**37**	Microtubule organizing center part GO:0044450	Cellular senescence GO:0090398	Thymic T cell selection GO:0045061
**38**	Regulation of interleukin 2 biosynthetic process GO:0045076	Natural killer cell mediated immunity GO:0002228	B cell mediated immunity GO:0019724
**39**	Thymic T cell selection GO:0045061	Regulation of p38mapk cascade GO:1900744	Positive regulation of response to oxidative stress GO:1902884
**40**	B cell receptor signaling pathway GO:0050853	Adaptive immune response based on somatic recombination of immune receptors built from immunoglobulin superfamily domains GO:0002460	Intrinsic component of mitochondrial outer membrane GO:0031306
**41**	Positive regulation of B cell proliferation GO:0030890	Wound healing spreading of epidermal cells GO:0035313	Myeloid leukocyte mediated immunity GO:0002444
**42**	Golgi membrane GO:0000139	Trans Golgi network transport vesicle GO:0030140	Cytokine production involved in immune response GO:0002367
**43**	Regulation of humoral immune response GO:0002920	ERAD pathway GO:0036503	Sarcoplasmic reticulum membrane GO:0033017
**44**	Regulation of cytokine biosynthetic process GO:0042035	Positive regulation of natural killer cell mediated immunity GO:0002717	Regulation of humoral immune response GO:0002920
**45**	Myeloid leukocyte differentiation GO:0002573	Axon regeneration GO:0031103	Positive regulation of interleukin 10 production GO:0032733
**46**	Lymphocyte costimulation GO:0031294	Neutrophil mediated immunity GO:0002446	Regulation of interleukin 13 production GO:0032656
**47**	Complement activation GO:0006956	Regulation of DNA repair GO:0006282	Defense response to gram positive bacterium GO:0050830
**48**	Perinuclear region of cytoplasm GO:0048471	Trans Golgi network transport vesicle membrane GO:0012510	Mast cell granule GO:0042629
**49**	B cell mediated immunity GO:0019724	Leukocyte mediated immunity GO:0002443	Base excision repair GO:0006284
**50**	Positive regulation of chemokine production GO:0032722	COPI vesicle coat GO:0030126	Derlin 1 retrotranslocation complex GO:0036513
**51**	Regulation of chemokine production GO:0032642	Base excision repair GO:0006284	Complement activation alternative pathway GO:0006957
**52**	Positive regulation of DNA damage response signal transduction by p53 class mediator GO:0043517	Perk mediated unfolded protein response GO:0036499	Clathrin vesicle coat GO:0030125
**53**	Endocytic vesicle GO:0030139	Cellular response to heat GO:0034605	Regulation of tumor necrosis factor biosynthetic process GO:0042534
**54**	Cytosolic part GO:0044445	Vesicle coat GO:0030120	Positive regulation of transcription from RNA polymerase ii promoter in response to endoplasmic reticulum stress GO:1990440
**55**	Vacuole GO:0005773	Cytoplasmic mRNA processing body GO:0000932	Gamma tubulin complex GO:0000930
**56**	Golgi apparatus GO:0005794	Cellular extravasation GO:0045123	COPI coated vesicle membrane GO:0030663
**57**	Positive regulation of cytokine production involved in immune response GO:0002720	Endosome lumen GO:0031904	Endosome lumen GO:0031904
**58**	Inflammasome complex GO:0061702	Regulation of natural killer cell mediated immunity GO:0002715	ER to Golgi transport vesicle membrane GO:0012507
**59**	Cellular response to stress GO:0033554	ER to Golgi transport vesicle membrane GO:0012507	Response to pain GO:0048265
**60**	Regulation of defense response to virus by host GO:0050691	Blood coagulation fibrin clot formation GO:0072378	Negative regulation of interferon gamma production GO:0032689
**61**	Microbody membrane GO:0031903	Positive regulation of interleukin 6 secretion GO:2000778	Negative regulation of cytokine biosynthetic process GO:0042036
**62**	Regulation of interleukin 2 production GO:0032663	Negative regulation of hemopoiesis GO:1903707	COPI vesicle coat GO:0030126
**63**	Golgi apparatus part GO:0044431	T cell differentiation involved in immune response GO:0002292	Positive regulation of acute inflammatory response GO:0002675
**64**	Secretory granule GO:0030141	Defense response to gram positive bacterium GO:0050830	Negative regulation of T cell differentiation GO:0045581
**65**	Cytosolic large ribosomal subunit GO:0022625	Intrinsic component of mitochondrial membrane GO:0098573	Error prone translesion synthesis GO:0042276
**66**	Positive regulation of interleukin 2 production GO:0032743	Positive regulation of leukocyte migration GO:0002687	Negative regulation of DNA repair GO:0045738
**67**	Regulation of cellular response to stress GO:0080135	Positive regulation of leukocyte chemotaxis GO:0002690	Negative regulation of response to endoplasmic reticulum stress GO:1903573
**68**	Innate immune response GO:0045087	Platelet aggregation GO:0070527	Positive regulation of vascular endothelial growth factor production GO:0010575
**69**	Positive regulation of immune response GO:0050778	Clathrin coat GO:0030118	Regulation of lymphocyte chemotaxis GO:1901623
**70**	Adaptive immune response GO:0002250	Response to misfolded protein GO:0051788	Regulation of cellular response to hypoxia GO:1900037
**71**	Intrinsic component of Golgi membrane GO:0031228	Positive regulation of interleukin 10 production GO:0032733	Regulation of macrophage activation GO:0043030
**72**	Large ribosomal subunit GO:0015934	Clathrin coat of trans Golgi network vesicle GO:0030130	Defense response to fungus GO:0050832
**73**	Vacuolar lumen GO:0005775	Regulation of double strand break repair GO:2000779	Zymogen granule GO:0042588
**74**	Macrophage differentiation GO:0030225	Regulation of leukocyte chemotaxis GO:0002688	Cell cortex region GO:0099738
**75**	Activation of immune response GO:0002253	Regulation of macrophage activation GO:0043030	Regulation of double strand break repair GO:2000779
**76**	Regulation of interferon beta production GO:0032648	Regulation of leukocyte migration GO:0002685	Lysosomal lumen GO:0043202
**77**	Leukocyte migration GO:0050900	Regulation of endoplasmic reticulum stress induced intrinsic apoptotic signaling pathway GO:1902235	Recycling endosome membrane GO:0055038
**78**	Positive regulation of response to DNA damage stimulus GO:2001022	Clathrin vesicle coat GO:0030125	Toll like receptor 4 signaling pathway GO:0034142
**79**	Defense response to otherorganism GO:0098542	Myeloid cell activation involved in immune response GO:0002275	Retrograde protein transport ER to cytosol GO:0030970
**80**	Transport vesicle GO:0030133	Inflammasome complex GO:0061702	Positive regulation of macrophage activation GO:0043032
**81**	Positive regulation of immune effector process GO:0002699	Granulocyte differentiation GO:0030851	Innate immune response in mucosa GO:0002227
**82**	Response to water deprivation GO:0009414	Non recombinational repair GO:0000726	Granulocyte differentiation GO:0030851
**83**	Mitochondrion GO:0005739	Antimicrobial humoral response GO:0019730	Regulation of vascular endothelial growth factor production GO:0010574
**84**	Myeloid cell differentiation GO:0030099	Regulation of removal of superoxide radicals GO:2000121	Inflammasome complex GO:0061702
**85**	Regulation of macrophage activation GO:0043030	Autophagosome membrane GO:0000421	Endocytic vesicle lumen GO:0071682
**86**	Negative regulation of platelet activation GO:0010544	Negative regulation of wound healing GO:0061045	Negative regulation of humoral immune response GO:0002921
**87**	Regulation of response to DNA damage stimulus GO:2001020	Lymphocyte mediated immunity GO:0002449	Regulation of cellular extravasation GO:0002691
**88**	Immune effector process GO:0002252	Negative regulation of T cell receptor signaling pathway GO:0050860	Outer mitochondrial membrane protein complex GO:0098799
**89**	Endocytic vesicle membrane GO:0030666	Regulation of neutrophil migration GO:1902622	Regulation of fibrinolysis GO:0051917
**90**	Negative T cell selection GO:0043383	Rough endoplasmic reticulum membrane GO:0030867	B cell proliferation GO:0042100
**91**	Immune system process GO:0002376	Pre autophagosomal structure GO:0000407	Regulation of interleukin 8 secretion GO:2000482
**92**	Regulation of defense response to virus GO:0050688	Defense response to fungus GO:0050832	Hematopoietic stem cell proliferation GO:0071425
**93**	Negative regulation of innate immune response GO:0045824	Positive regulation of response to oxidative stress GO:1902884	PERK mediated unfolded protein response GO:0036499
**94**	Regulation of immune effector process GO:0002697	Intrinsic component of Golgi membrane GO:0031228	Regulation of megakaryocyte differentiation GO:0045652
**95**	Secretory vesicle GO:0099503	Error prone translesion synthesis GO:0042276	Response to misfolded protein GO:0051788
**96**	Positive regulation of B cell activation GO:0050871	Negative regulation of lymphocyte differentiation GO:0045620	Positive regulation of interleukin 8 secretion GO:2000484
**97**	Early endosome membrane GO:0031901	Regulation of cellular extravasation GO:0002691	Mast cell mediated immunity GO:0002448
**98**	Cytosolic ribosome GO:0022626	Golgi lumen GO:0005796	Response to immobilization stress GO:0035902
**99**	Cellular response to glucose starvation GO:0042149	Recycling endosome membrane GO:0055038	Negative regulation of alpha beta T cell activation GO:0046636
**100**	Centriole GO:0005814	Regulation of Toll like receptor signaling pathway GO:0034121	Mature B cell differentiation involved in immune response GO:0002313
**101**	Regulation of alpha beta T cell differentiation GO:0046637	Hyperosmotic response GO:0006972	Mitotic G2 DNA damage checkpoint GO:0007095
**102**	Regulation of immune response GO:0050776	COPI coated vesicle membrane GO:0030663	Negative regulation of alpha beta T cell differentiation GO:0046639
**103**	Clathrin coated vesicle GO:0030136	Somatic recombination of immunoglobulin gene segments GO:0016447	Tricarboxylic acid cycle enzyme complex GO:0045239
**104**	DNA damage response detection of DNA damage GO:0042769	Neuron projection regeneration GO:0031102	Positive regulation of monocyte chemotaxis GO:0090026
**105**	Regulation of adaptive immune response GO:0002819	Clathrin coat of endocytic vesicle GO:0030128	Wash complex GO:0071203
**106**	Ribosome GO:0005840	Erythrocyte maturation GO:0043249	Regulation of removal of superoxide radicals GO:2000121
**107**	Regulation of B cell proliferation GO:0030888	Hemoglobin complex GO:0005833	Protein phosphatase type 1 complex GO:0000164
**108**	Mitochondrial part GO:0044429	Response to pain GO:0048265	Bloc complex GO:0031082
**109**	Regulation of type I interferon production GO:0032479	B cell homeostasis GO:0001782	Negative regulation of platelet activation GO:0010544
**110**	Activation of JUN kinase activity GO:0007257	Negative regulation of interferon gamma production GO:0032689	Regulation of chemokine biosynthetic process GO:0045073
**111**	Positive regulation of lymphocyte differentiation GO:0045621	Response to axon injury GO:0048678	Regulation of regulatory T cell differentiation GO:0045589
**112**	Regulation of macrophage differentiation GO:0045649	Negative regulation of interleukin 6 production GO:0032715	Hemoglobin complex GO:0005833
**113**	Positive regulation of type 2 immune response GO:0002830	B cell proliferation GO:0042100	Negative regulation of endoplasmic reticulum unfolded protein response GO:1900102
**114**	Regulation of B cell activation GO:0050864	Negative regulation of alpha beta T cell activation GO:0046636	Negative regulation of Toll like receptor signaling pathway GO:0034122

### GO tree analysis of the relationship between deregulated immune/inflammation functions

**Figure 3:**
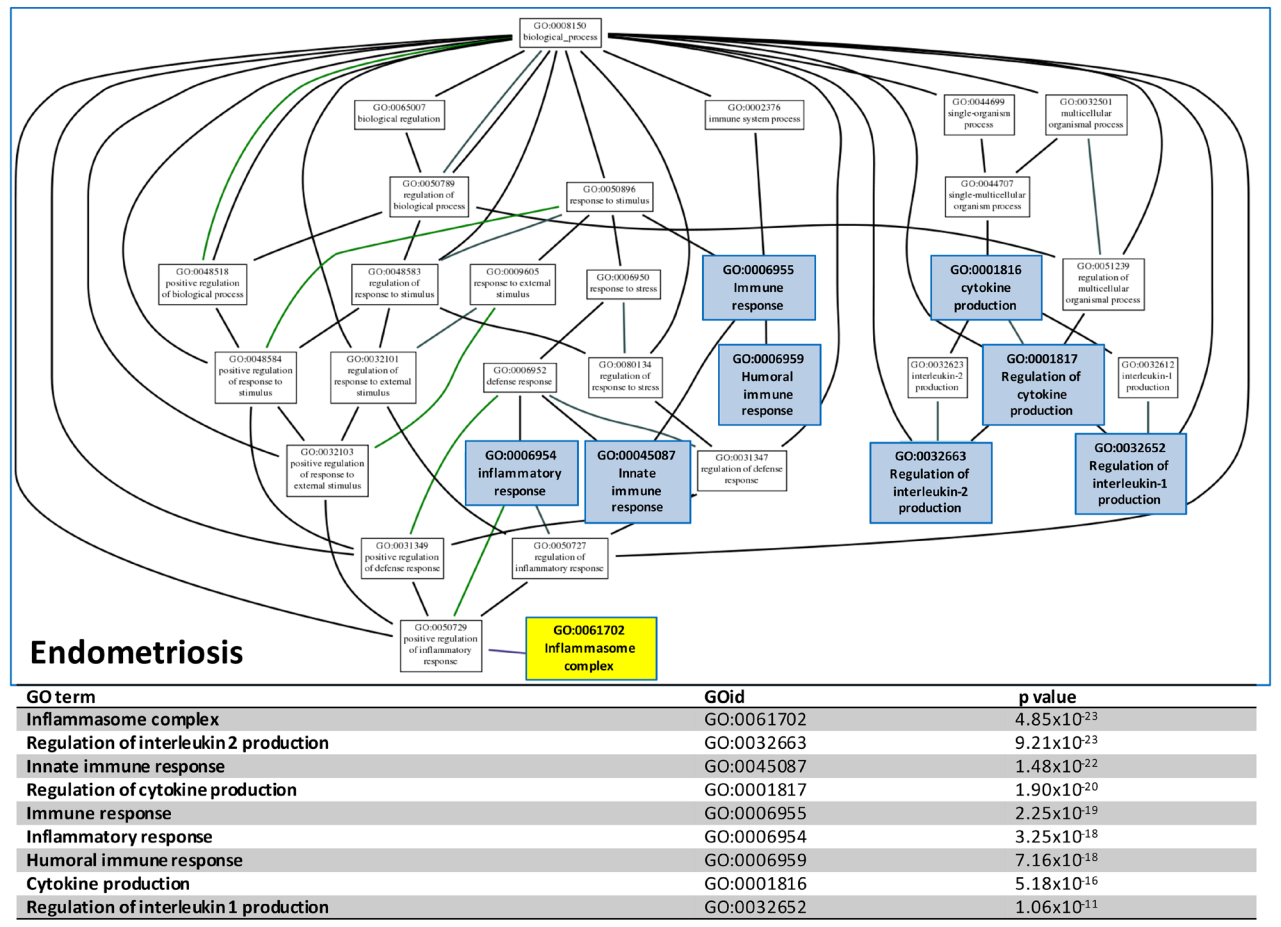
GO tree analysis The GO tree of deregulated functions of CCC establish with the significant GO terms involving in the inflammation and immune system. After mapping to the GO tree, the similar or related GO terms were clustered together and shown the parent-child relationship. The table listed the immune or inflammation-related GO terms, the GOIDs and their p values in the GO trees.

To concentrate and view the hierarchy of the numerous identified deregulated GO terms, we mapped the immune/inflammation related GO terms to the GO tree based on the parent-child relationship. The related GO terms on the GO tree were then clustered together so the relationship of these GO terms can be visualized and summarized up as Figure 3 shown. The deregulated functions on the GO trees for ES could be summarized to ‘immune response’, ‘inflammation response’, ‘cytokine production’ and ‘inflammasome complex’. The inflammasome complex was highlighted because it was known to be an activator the carcinogenesis in many cancers. The full GO trees of the three diseases are available in Supplementary Figure 2-4. The data-mining approach above revealed the inflammasome complex was one of the most crucial candidate function initiating the malignant transformation of EAOC. In order to discover the genes involving in the inflammasome complex for further investigation and confirmation, we carried out an integrative analysis using the same microarray gene expression datasets to detect the differentially expressed genes (DEGs) of the three diseases. All of the gene expressions of the samples in each dataset were rescaled to the cumulative proportion for the integrative analysis. The full table of the DEGs is available in Supplementary Table 4. We then filtered the genes that were related to inflammasome complex. This filtering obtained a list of 47 genes, as shown in Supplementary Table 5.

### Expression of inflammasome complex and inflammasome-related genes correlate with poor survival outcome in EAOC patients

**Figure 4:**
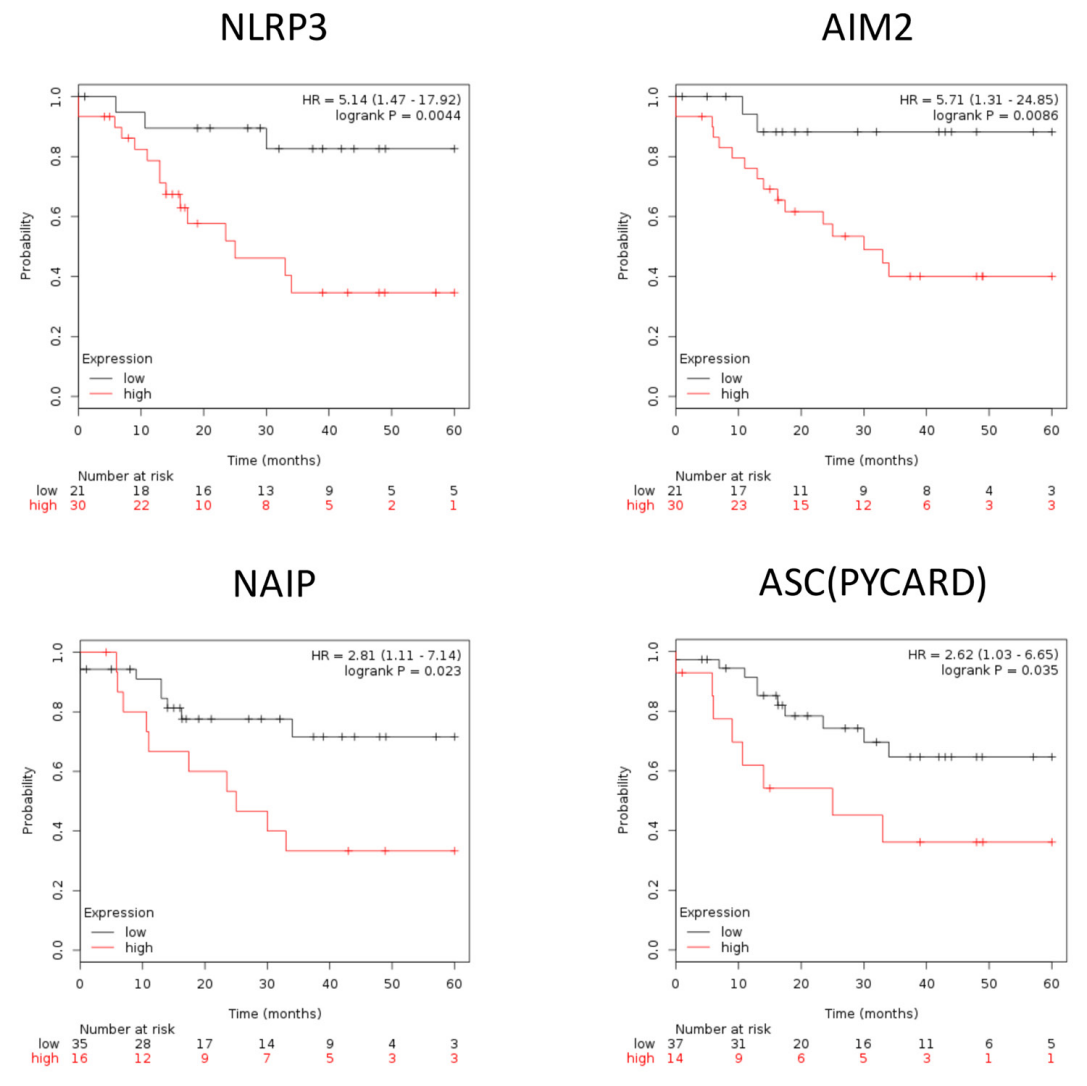
Inflammasome complex correlate with survival outcome in EAOC patients Kaplan–Meier plotter survival curves showed significant difference of EAOC survival with different expression level of inflammasome complex (NLRP3, AIM2, PYCARD, NAIP). HR = 5.14, 95% CI 1.47 to 17.92, *p*-value = 0.044; HR = 5.71, 95% CI 1.31 to 24.85, *p*-value = 0.086; HR = 2.62, 95% CI 1.03 to 6.65, *p*-value = 0.035; HR = 2.81, 95% CI 1.11 to 7.14, *p*-value = 0.023, respectively.

**Figure 5:**
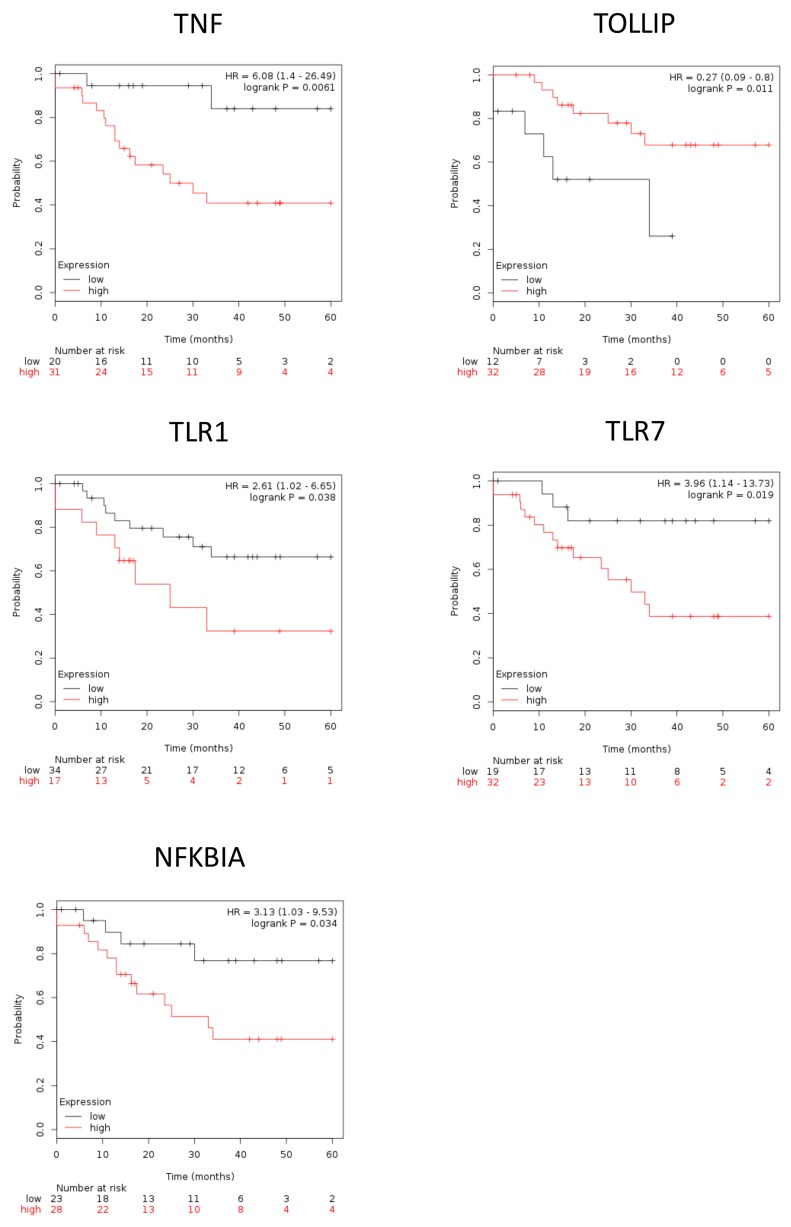
The survival of EAOC patients are correlate with inflammasome-related genes Kaplan–Meier plotter survival curves showed significant difference of EAOC survival with different expression level of inflammasome-related genes (TNF, FOXO3, TLR7, NFKBIA). HR = 6.08, 95% CI 1.4 to 26.49, *p*-value = 0.061; HR = 3.15, 95% CI 1.24 to 8.02, *p*-value = 0.011; HR = 3.96, 95% CI 1.14 to 13.73, *p*-value = 0.019; HR = 3.13, 95% CI 1.03 to 9.53, *p*-value = 0.034, respectively.

To further illustrate the role of inflammasome in EAOC progression, we used Kaplan–Meier plotter (http://www.kmplot.com/ovar) to explore the correlation between EAOC patient survival and the expression levels of inflammasome complex as well as inflammasome-related geneses. Inflammasomes are multimeric protein complexes. Activation of inflammasomes and regulation of related pathway capable of orchestrating host inflammation and immunity [15, 16]. The component of inflammasome in tumorigenesis included inflammasome complex and inflammasome-related pathway [17, 18]. Inflammasome complex included nucleotide-binding domain and leucine-rich repeat receptors (NLRs), absent in melanoma 2(AIM2) and apoptosis*-*associated speck-like protein containing a CARD (ASC). NLRs and AIM2 recruit pro-caspase and promote its autocatalytic cleavage into active caspase, which leads to a cascade of pro-inflammatory events via the activation of the pro-inflammatory cytokine, which then interacts with their membranes receptors (TLR, TNF, INF, P2RX7) and related pathway amplifying the inflammatory response. We checked the 47 genes in Supplementary Table 5; they included 7 genes of inflammasome complex (NLRP3, AIM2, PYCARD, NAIP, Caspase-4, Caspase-7 and Caspase-8) and 11 genes of the inflammasome-related pathway (TLR1, TLR7, TOLLIP, NFKBIA, TNF, TNFAIP3, INFGR2, P2RX7, IL-1B, IL1RL1 and IL-18). Based on a database created by Gyorffy et al. [19], we correlated the gene expression levels of 18 highly expressed inflammasome markers, including 7 inflammasome complex genes and 11 inflammasome genes related pathway, with EAOC patient survival outcome. We found that high expression levels of the 7 inflammasome complex genes (NLRP3, AIM2, PYCARD, NAIP, Caspase-4, Caspase-7 and Caspase-8) tend to correlate with poor patient survival, and four of them (NLRP3, AIM2, PYCARD, NAIP) were statistically significant (Figure 4). NLRP3 and AIM2 are the initiators of inflammasomes, while PYCARD and NAIP are the core proteins of inflammasomes. These results indicated a potential direct involvement of inflammasome in EAOC progression. In the 11 genes inflammasome-related pathway (TLR1, TLR7, TOLLIP, NFKBIA, TNF, TNFAIP3, INFGR2, P2RX7, IL-1B, IL1RL1 and IL-18), high expression of these genes tended to correlate with poor survival of EAOC patients and 5 of them (TLR1, TLR7, TOLLIP, NFKBIA and TNF) reached statistical significance (Figure 5). The other 9 inflammasome-related genes (Caspase-4, Caspase-7, Caspase-8, TNFAIP3, INFGR2, P2RX7, IL-1B, IL1RL1 and IL-18) were not correlate with survival of the EAOC patients (Supplementary Figure 5-6). The flowchart and selection criteria of the EAOC marker genes were demonstrated as Supplementary Figure 7. These results indicated the involvements of inflammasome complex and inflammasome-related pathways in mediating EAOC disease progression.

Notably, the survival outcome of EAOC patients was highly correlated with NLRP3, AIM2, and TNF. The hazard ratio of NLRP3 / AIM2 / TNF were 5.14(1.47-17.92) / 5.71(1.31-24.85) / 6.08(1.4-26.49), respectively; p = 0.0044 / 0.0086 / 0.0061, respectively) (Figure 4 and 5). These results suggested key roles of the three inflammasome proteins and related pathways in promoting EAOC progression as well as their prognostic value in EAOC.

**Figure 6:**
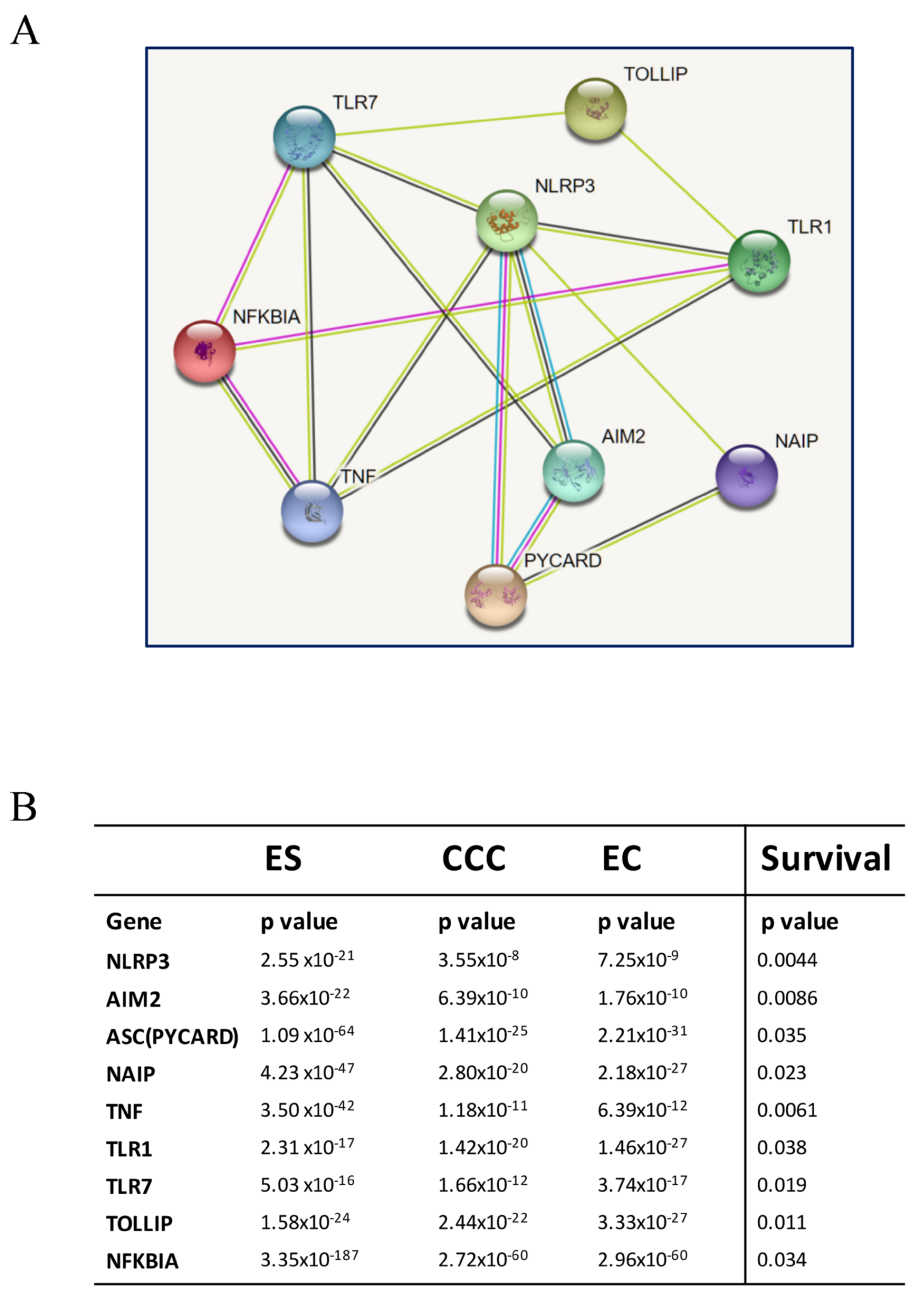
Interaction analysis of identified genes **(A)** The identified potential involving genes were subjected to a protein-protein interaction (PPI) analysis by establishing an interactive network from the STRING database (https://string-db.org). As members of inflammasome complex and inflammasome-related genes, their proteins showed intensive interactions. The average node degree is 3.56, and the PPI enrichment p-value is 3.33x10^-15^, significantly more interactions than expected. **(B)** The p values of each gene in the three diseases were showed in the chart. The progressive changes of p values from ES to CCC and EC demonstrated that the NLRP3, AIM2, PYCARD, NAIP, TLR7, NFKBIA, TNF, FOXO3 would be the potential markers of prognosis in EAOC.

Based on the survival analysis (Figure 4 and 5), we used the 9 inflammasome markers and STRING database (https://string-db.org) to establish a functional interaction network (Figure 6A). As members of inflammasome complex and inflammasome pathway related genes, the 9 proteins showed intensive interactions and regulatory crosstalk. This interactive network supported the involvement and key role of inflammation in EAOC malignant progression. Collectively, we demonstrated that the NLRP3, AIM2, PYCARD, NAIP, TLR1, TLR7, TOLLIP, NFKBIA and TNF would be the potential markers of prognosis in EAOC (Figure 6B).

### Immunohistochemistrical analysis for AIM2 expression among the three diseases

**Figure 7:**
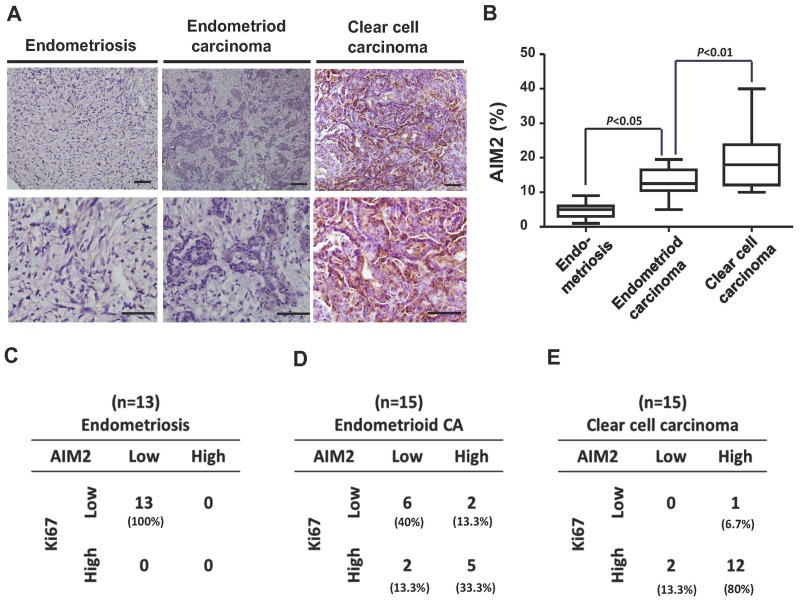
Immunohistochemistrical analysis of clinical samples from patients with ES, EC, and CCC **(A)** Clinical samples from patients with ES (n = 13), EC (n = 15), and CCC (n = 15) were immunostained with anti-AIM2 antibody. **(B)** The expression levels of AIM2 in all clinical samples were quantified and presented in the chart. The mean values of AIM2 expression in EC and CCC were higher than that in ES. **(C-E)** Samples were stained with Ki-67 and AIM2. The case numbers of ES, EC, and CCC with high and low expression levels of Ki-67 and AIM2 were calculated and displayed in the chart. The percentages of each combination were also calculated. The AIM2 levels was positively correlated with Ki-67 levels.

To evaluate the clinical significance of the identified inflammasome-related genes in ovarian cancer transformation, we collected a cohort of clinical samples (ES, n = 13; CCC, n = 15; EC, n = 15) and immunostained them with anti-AIM2 antibody. We found increased AIM2 protein level in CCC and EC samples in comparison to ES samples (Figure 7A). Quantification of AIM2 levels in all samples showed a higher mean value of AIM2 protein expression in both cancer types than in ES (Figure 7B). We then calculated the case numbers of AIM2-high and AIM2-low, as well as Ki-67-high and Ki-67-low in ES, CCC, and EC samples, and correlated the status of the two markers in each type of samples. As shown in Figure 7C–7E, we generally observed a positive correlation between the expression levels of Ki-67 and AIM2. In the 13 ES samples, all of them exhibited only low levels of Ki-67 and AIM2 (Figure 7C), while in the CCC samples, 12 out of 15 expressed high levels of Ki-67 and AIM2 (Figure 7E). In the group of EC, 5 samples expressed high Ki-67 and AIM2 level, and 6 expressed low levels of the two proteins (Figure 7D). Calculation of the percentage of AIM2-high case showed a progressive increase from ES to EC and to CCC (Figure 7C–7E). These results provid clinical evidence supporting the involvement of AIM2 in the malignant transformation of ES to CCC/EC.

### Working model of inflammasome in endometriosis associated ovarian carcinoma

**Figure 8:**
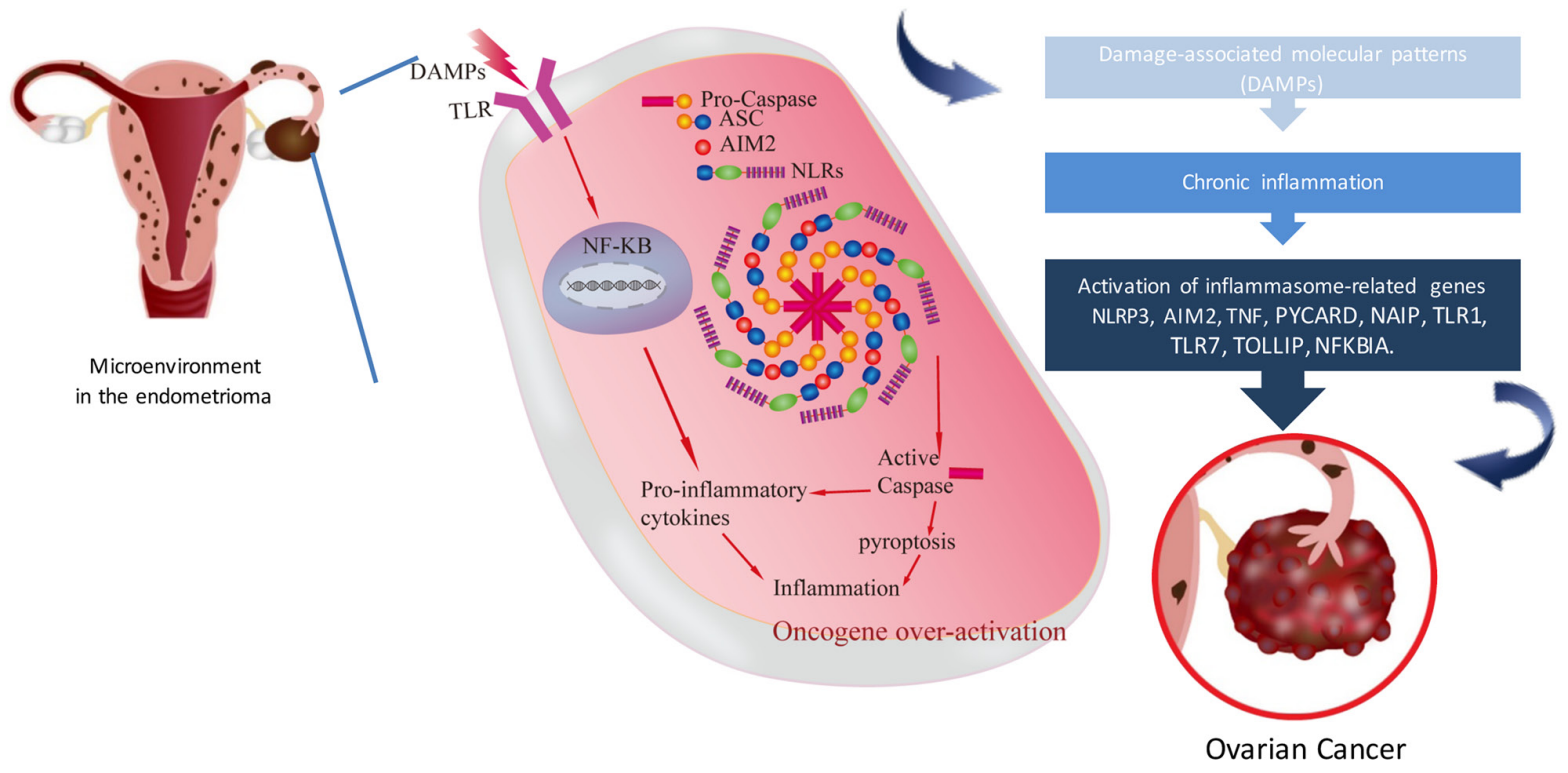
Working model of the inflammasome in endometriosis associated ovarian cancer This model presents the microenvironment in endometrioma of the ovary. Retrograded menstruation accumulated in ovary provoked DAMPs and caused chronic inflammation. Inflammasome related genes (NLRP3, AIM2, PYCARD, NAIP, TNF, FOXO3, TLR7, NFKBIA) were activated subsequently. Activated caspase can lead to cell pyroptosis with the consequence of the release of inflammatory cytokines. Finally, inflammatory cytokines induced oncogene over-expression then produced EAOC carcinogenesis.

Based on our data-driven analysis and lab validation, we proposed a working model of the association between inflammasome in endometriosis and the progression of ovarian cancer. In the microenvironment of ovarian endometrioma, inflammasome is driven directly by specific DAMPs or by the two-signal model as in the case of NLRP3 in the microenvironment of ovarian endometrioma, The recognition of DAMPs by extracellular TLRs leads to the activation of NF-κB (first signal), which in turn promotes the transcription of pro- inflammatory cytokines or some NLRs (e.g.NLRP3). NLRs assemble into the inflammasome complex which via the CARD domain can recruit pro-caspase and promote its autocatalytic cleavage (second signal). Active caspase can lead to a cascade of pro-inflammatory events via the activation of pro- inflammatory cytokines, which then interact with their own membrane receptors amplifying the inflammatory response. On the other hand, active caspase can lead to cell pyroptosis with the consequence of the release of inflammatory cytokines. Inflammatory cytokines activated oncogene over-expression then induced EAOC carcinogenesis (Figure 8).

## DISCUSSION

Complex diseases usually involve in a spectrum of variable deregulated functions. So we investigated the pathogenesis of EAOC with the functionome consisted of 5917 GO defined functions computed from large-scale microarray gene expression profiles. We demonstrated the informativeness of the GSR indices was sufficient for machine learning to accurately recognize and classify these complex diseases based on the functional regularity patterns. The patterns were similar between CCC and EC as showed on the heatmap (Figure 2), revealing the possibility of homogeneous etiology between these two cancers. We further investigated the common deregulated functions among ES, CCC, and EC to discover the candidate elements involving in the malignant transformation from ES to CCC or EC. Our study revealed the consistent findings: the ‘activation of immune response’ in ES; the ‘humoral immune response’ deregulated GO terms for CCC and EC. Moreover, the deregulated GO term ‘inflammatory response’ (GO:0006954) coexisted in ES, CCC, and EC. We further checked the immune/inflammation related GO terms in the functionomes of the three diseases. The set analysis using the top significant 114 immunes/inflammation related GO terms for the three diseases showed nine common deregulated GO terms, and the existence of inflammasome complex in this list is noticeable because it has been demonstrated to be a critical promoter of carcinogenesis in various cancers. Then we checked the DEGs detected from the same DNA microarray datasets, the inflammasome related genes, including NLRP3, AIM2, PYCARD, NAIP, TLR1, TLR7, TOLLIP, NFKBIA and TNF were demonstrated to be differentially expressed in the three diseases and also significantly correlated with poor progression-free survival. Finally, high expressions level of AIM2 were confirmed in EC and CCC, in comparison to ES, by immunohistochemical analysis, and is correlated with high level of Ki-67. Our results support that a close relationship between endometriosis and clear cell carcinoma/endometrioid carcinoma, and support the hypothesis of endometriosis associated ovarian carcinoma. Dysregulated inflammasome could be a fundamental role in modulating the malignant transformation of EAOC, which also broadens the scope of the inflammation/immunity as a molecular biomarker in monitoring the malignant transformation of endometriosis and also could be the treatment target of endometriosis associated ovarian cancer. To the best of our knowledge, these findings are vital to clarify the role of the inflammasome in EAOC carcinogenesis.

The inflammatory microenvironment has been revealed to play crucial roles in all stages of tumor development [20]. Pathogen or damage signals that trigger inflammation have been reported to drive tumorigenesis in many forms of cancer [11]; immune cells that trigger inflammation were also associated with tumor development [21]. The immune microenvironment is critical for the carcinogenesis of EAOC. The cell proliferation resulted from aberrations humoral immunity and complement pathway activation was postulated to play a major role in the pathogenesis of EAOC [22]. Cancer-immune phenotypes in humans can be divided into three main categories: the immune-desert phenotype, the immune–excluded phenotype and the inflamed phenotype. Each is related to specific underlying biological mechanisms that may prevent the host’s immune response from eliminating cancer. Inflamed tumors are infiltrated by a variety subtypes of immune cells including immune-inhibitory regulatory T cells, myeloid-derived suppressor cells, and cancer-associated fibroblasts [23]. The presence of intratumoral T cells independently associated with delayed recurrence or prolonged survival in multivariate analysis of advanced ovarian carcinoma and was related to increased expression of interferon-γ, interleukin-2, and lymphocyte-attracting chemokines within the tumor [24]. Anti-inflammatory effects in autoimmune diseases and neurodegeneration also appeared to suppress the inflammatory activity of TLR4-NF-κB/ NLRP3 inflammasome pathway and provided novel mechanistic insights for the potential therapeutic for cervical cancer [25]. The inflammasome of NOD-like receptor family pyrin domain-containing 3 (NLRP3) is a complex protein involved in the induction of innate inflammatory/immune responses. The complex consists of the NLRP3 protein, which serve as a sensor for the activation of the inflammasome, and an apoptosis-associated speck-like protein containing a CARD complex (ASC), which recruits pro-caspase through its CARD domain. Pro-caspase is then interchange to active caspase, which, in turn, cleaves pro-inflammatory cytokines (pro-IL-1β and pro-IL-18) to their active forms. IL-1β and IL-18 to promote inflammation by recruiting additional inflammatory/immune cells. Then oncogene could be activated. Thus, NLRP3 signaling persistent sterile inflammation could be the initial stage of carcinogenesis.

AIM (absent in melanoma 2) can induce inflammasome upon intracellularly delivery of double-stranded DNA (dsDNA) to protect cells against pathogens like virus and bacteria. AIM2 is a cytosolic dsDNA sensor and directly interacts with dsDNA, mainly from virus or bacteria, through its C-terminal HIN-200 domain, leading to a serial activation of inflammation proteins and form AIM2 inflammasome. Activation of the AIM2 inflammasome and other canonical inflammasomes results in a type of inflammatory cell death called pyroptosis. Chronic inflammation of the benign prostate hyperplasia was reported closely related to prostate cancer. Recent studies showed that AIM2 inflammasome plays a critial roles in the tumor progression of prostate cancer. Activation of AIM2 was served as a biomarker to identify the molecular mechanisms through prostatic infections and/or sterile inflammation contribute to the carcinogenesis of prostatic cancer [26]. In our comparative bioinformatic analysis between endometriosis and ovarian carcinoma, we found AIM2 diversely expressed in the two groups of data, suggesting a role of AIM2 in promoting the progression of ovarian carcinoma. Notably, the analysis of immunohistochemistry staining further confirmed a correlation between high AIM2 expression and high Ki-67 activity in clinical EAOC samples, supporting that AIM2 and inflammasome play a key regulatory role in EAOC transformation and disease progression. Therefore, based on our findings, inflammation mechanism is suggested as the key regulatory step mediated the malignant transformation of endometriosis. Further *in vivo* study to investigate whether NLRP3/AIM2 contributes to EAOC carcinogenesis and the role of the inflammasome in EAOC is imperative.

This investigation has limitations, though. First, the GO term gene set database does not define the comphehensive human functions yet. Therefore, undefined immulogical functions involving in the malignant transformation may be missed in the current analysis. Second, the GSR model may produce false positive results because of similar gene elements in different gene sets. For example, the 47^th^ desregulated functions for EC ‘Defense response to gram positive bacterium (GO:0050830)’ in the Table 3 may potentially be a false positivity; because to our knowledge, there is no evidence showing the involvement of gram positive vacterial infection in the etiology of EC. It may raise from the duplicated gene elements in the gene set definitions. Third, the case number for the immunohistochemistrical analysis is relatively small. More cases are necessary to clarify the pathogenesis of EAOC in the future.

In conclusion, we established a bioinformatic platform of gene-set integrative molecular functionome to dissect the molecular pathogenetic pathways of EAOC and demonstrated dysregulated inflammasomes play a fundamental role in modulating the malignant transformation and cancer progression in EAOC. Our results support the hypothesis that endometriosis shares similar genetic signatures with EAOC that validated by data-driven analysis and tissue array, which also broaden the scope of the inflammation/immunity as a molecular biomarker in monitoring the malignant transformation of endometriosis and also could be the treatment target of endometriosis associated ovarian cancer.

## MATERIALS AND METHODS

### Computing the GSR indices

The regulation of the GO terms were quantified by the GSR model, which converted gene expression profiles to quantified functions by the modifying the Differential Rank Conservation (DIRAC) [27] algorithm. This model quantifies the ordering change of the gene elements in a gene set between the gene expression orderings in ES, CCC or EC and the most common gene expression ordering in the normal control population in this study. Microarray gene expression profiles were downloaded from the Gene Expression Omnibus (GEO) database as.SOFT format, and then the gene expression levels were extracted according to the corresponding gene elements in the GO term gene set and converted to the ordinal data based on their expression levels. The GSR index is the ratio of gene expression ordering in a gene set between each case or normal control sample and the most common gene expression ordering among the normal tissue samples. Computing the GSR indices was executed in R environment. The detail of the GSR model and the computing procedures are described in our previous study [21].

### Gene set definition, microarray datasets and data processing

The versions of the GO gene set definitions were c5.all.v6.0.symbols.gmt (2017), downloaded from the MSigDB and contained 5917 GO gene sets. The selection criteria for the downloaded microarray gene expression datasets were: 1. The datasets should provide definite information on the diagnosis for each sample; 2. Because this study utilized the common genes among the selected datasets; a dataset was discarded if it resulted in the number of common genes less than 8000 when it was integrated.

### Statistical analysis

The differences of the GSR indices between the three diseases and the control groups were tested by Mann Whitney U test and corrected by multiple hypotheses using false discovery rate (Benjamini-Hochberg procedure). The significance level was set at <0.01. Progression-free survival (PFS) data of endometrioid ovarian cancer were available for 51 patients obtained from kmplot.com. The Kaplan–Meier survival curves for endometrioid ovarian cancer can be reached at http://www.kmplot.com/ovar. Hazard ratio (HR; and 95% confidence intervals) and logrank P are calculated and displayed with website.

### Classification and prediction by machine learning

GSR indices computed through the GO term gene sets were classified and predicted by the support vector machine (SVM) with kernlab [28], an R package for kernel-based machine learning methods and was used to classify patterns of the GSR indices with the setting of kernel = ‘vanilladot’ (linear kernel function). The performance of classification and prediction by SVM were measured by 5-fold cross-validation: samples were randomly sampled and divided into 5 parts, 4 parts were used for training sets and the remainder one part for prediction. The performance of binary classification was assessed with sensitivity, specificity, accuracy and AUC. Sensitivity, specificity, accuracy and AUC were computed using the results of successive 10 classifications. AUC was computed by an R package pROC [29]. The accuracy of multiclass classification was computed from the fraction of correct predictions within total prediction number.

### Hierarchical clustering, dendrogram and heatmaps

The GSR indices in each gene set were averaged then underwent hierarchical clustering with the function ‘heatmap.2’ in R package ‘gplots’ (version 3.0.1) as default. This function executed hierarchical clustering, and drew dendrogram and heatmaps.

All possible logical relations among the deregulated functions of the three diseases were evaluated and displayed by Venn diagram using the R package ‘VennDiagram’ (version 1.6.17).

### Reconstruction of GO trees and detection of differentially expressed genes

The GO tree were reconstructed by the ‘RamiGO’ [30], an R package providing functions to interact with the AmiGO 2 web server (http://amigo2.berkeleybop.org/amigo) and retrieves GO trees. To discover the DEGs for each of ES, CCC and EC, we carried out an integrative analysis with the same DNA microarray datasets. The gene expression levels of all samples in each dataset were transformed and rescaled to cumulative proportion values from 0 (lowest expression) to 1 (highest expression) with an R package “YuGene” (version 1.1.5) before integration. The DEGs were discovered using linear model computed with empirical Bayes analysis by the functions “lmFit” and “eBayes” provided by the R package “limna” (version 3.26.9).

### Clinical samples

The present study included 30 archived ovarian cancers (clear cell, N = 15 and endometrioid, N = 15), 13 ovarian endometrioma. In the cases of ovarian cancer, tissues were collected from women underwent surgery as their treatment guideline, and tissue specimens of endometrioma were collected from women who had ovarian endometrioma undergone a surgery of ovarian cystectomy. The patients were diagnosed and treated and had their tissues placed in a bank at the Tri-Service General Hospital, Taipei, Taiwan. All invasive cancers were confirmed by histopathology. The Institutional Review Board of the Tri-Service General Hospital, National Defense Medical Center approved the study. Informed consent was acquired from all patients and control subjects.

## SUPPLEMENTARY MATERIALS FIGURES AND TABLES












