# Supplementary material for: Integrating the dysregulated inflammasome-based molecular functionome in the malignant transformation of endometriosis-associated ovarian carcinoma
Source: Oncotarget. 2017 Dec 18;9(3):3704–26. doi: 10.18632/oncotarget.23364 (PMC5790494; doi:10.18632/oncotarget.23364)
Supplement: Supplementary file 1 [file oncotarget-09-3704-s001.pdf]

# Integrating the dysregulated inflammasome-based molecular functionome in the malignant transformation of endometriosis-associated ovarian carcinoma

## SUPPLEMENTARY MATERIALS

**Supplementary Table 1: Table of the sample information listing the phenotypes, DNA microarray platforms, FTP and GEO accession numbers of the study samples.**

**See Supplementary File 1**

**Supplementary Table 2: The complete GO terms of the three diseases ordered by the P values.**

**See Supplementary File 2**

**Supplementary Table 3: The 114 deregulated immune/inflammation related GO terms in the endometriosis and EAOC.**

**See Supplementary File 3**

**Supplementary Table 4: Total DEGs in endometriosis and EAOC.**

**See Supplementary File 4**

**Supplementary Table 5: The DEGs of 47 genes related to inflammasome.**

**See Supplementary File 5**

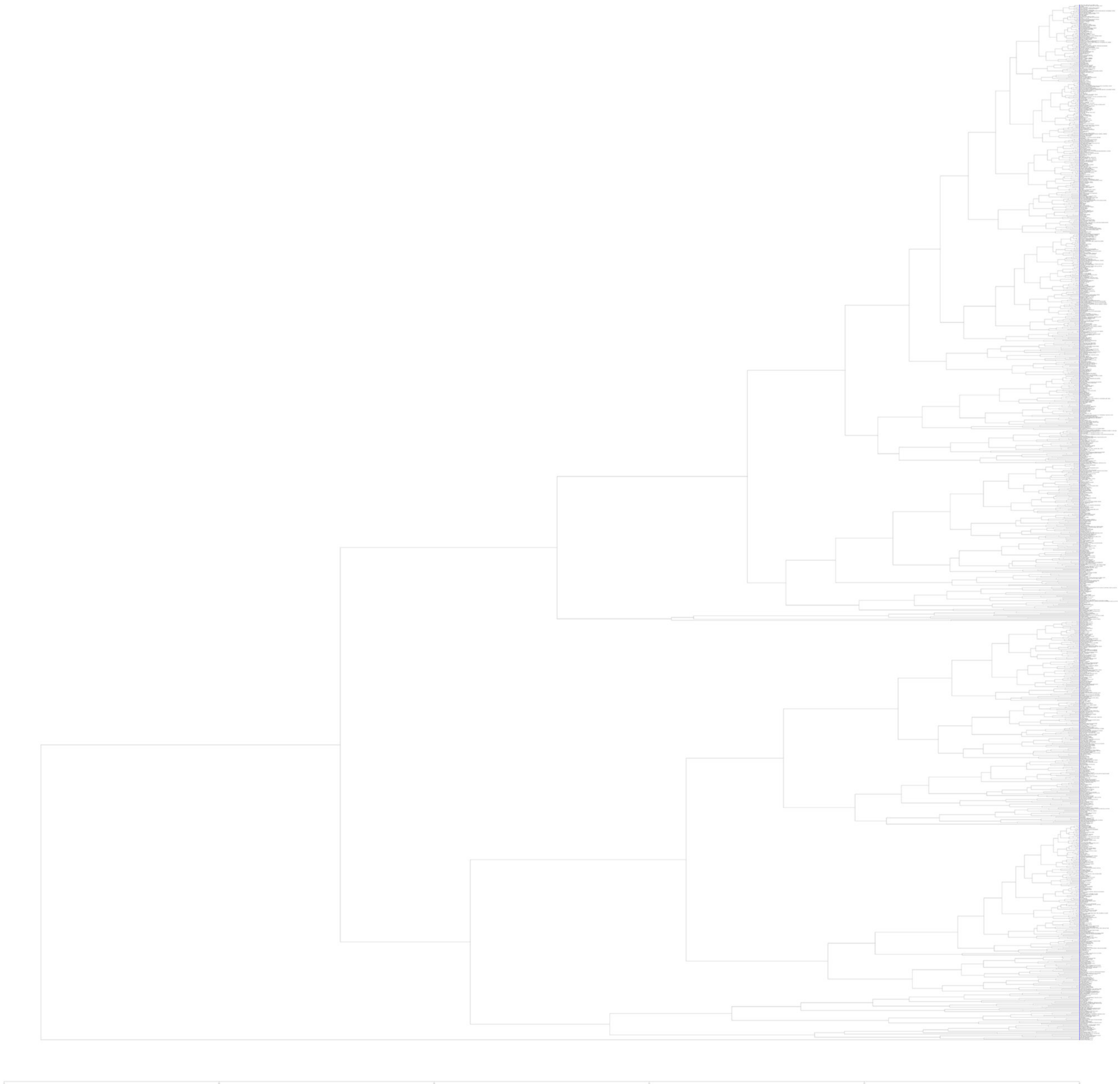

**Supplementary Figure 1: Full figure of the dendrogram for endometriosis and EAOC.**

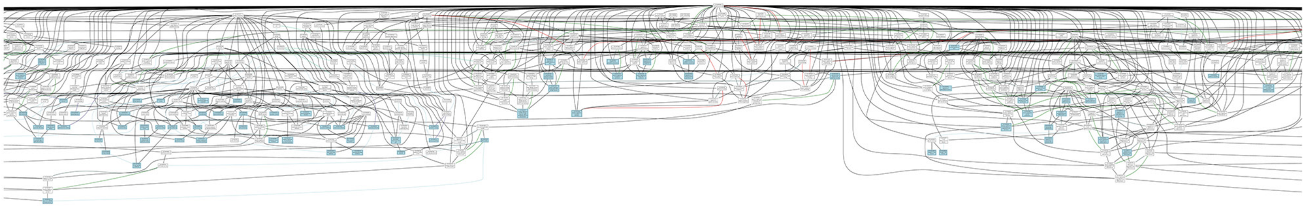

**Supplementary Figure 2: Full figure of the GO tree for endometriosis.**

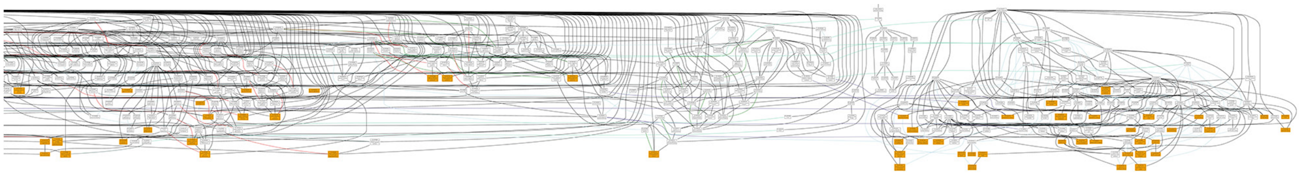

**Supplementary Figure 3: Full figure of the GO tree for ovarian clear cell carcinoma.**

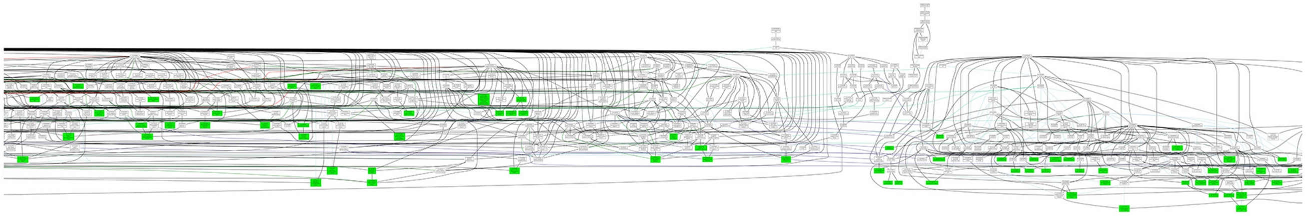

**Supplementary Figure 4: Full figure of the GO tree for ovarian endometrioid carcinoma interaction network for endometriosis associated ovarian carcinoma.**

CASPASE-4

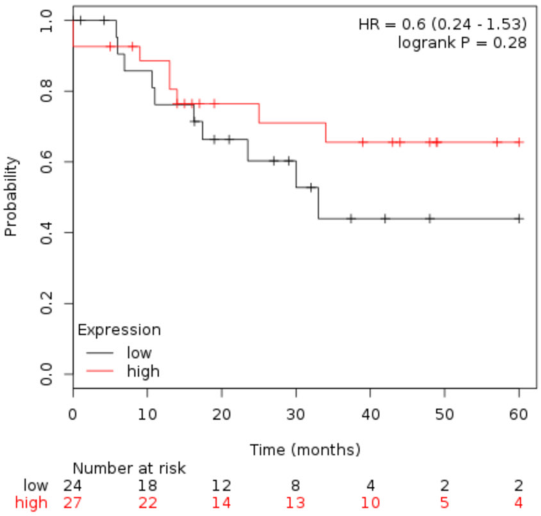


CASPASE-7

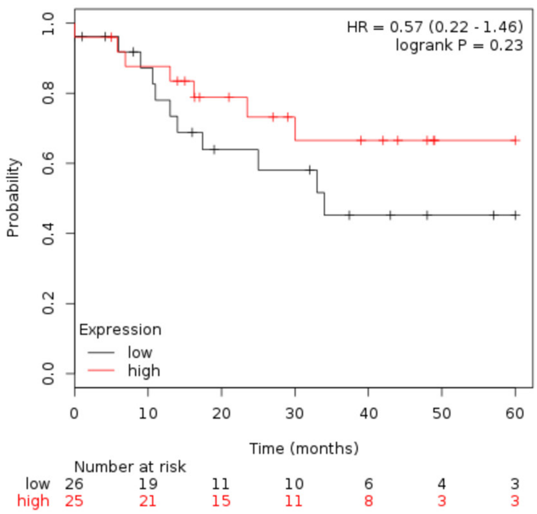


CASPASE-8

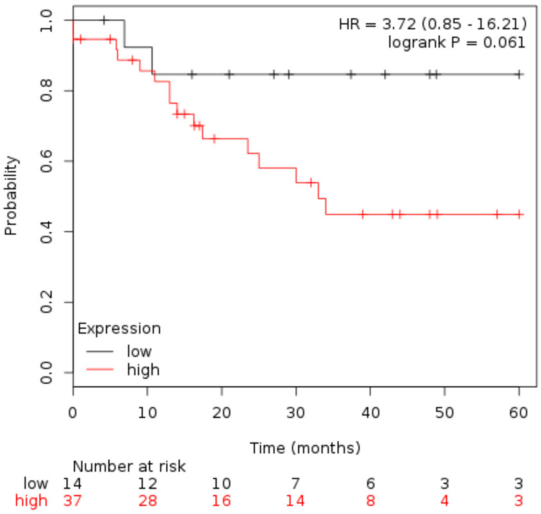


**Supplementary Figure 5: Inflammasome complex genes not correlate with survival outcome in EAOC patients.**

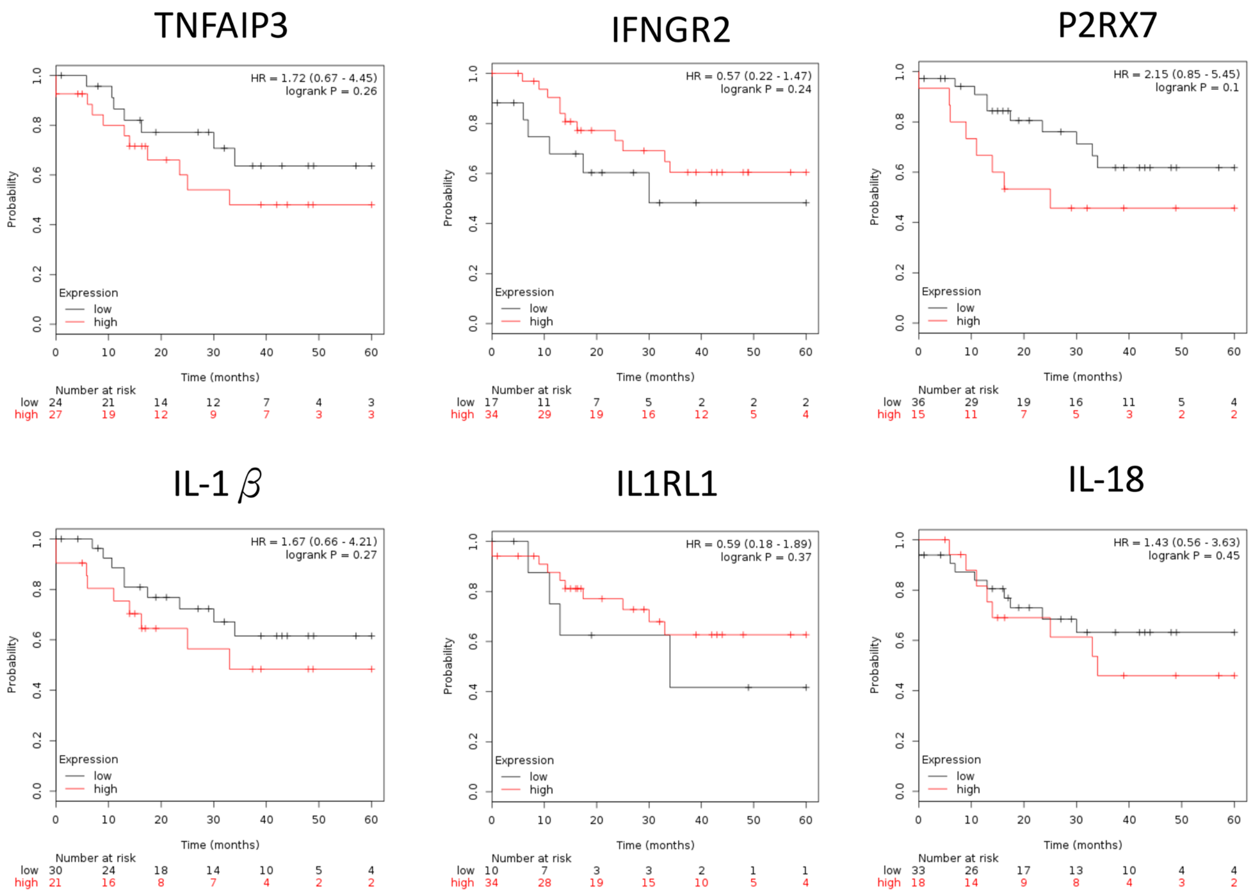


**Supplementary Figure 6: Inflammasome-related genes not correlate with survival outcome in EAOC patients.**

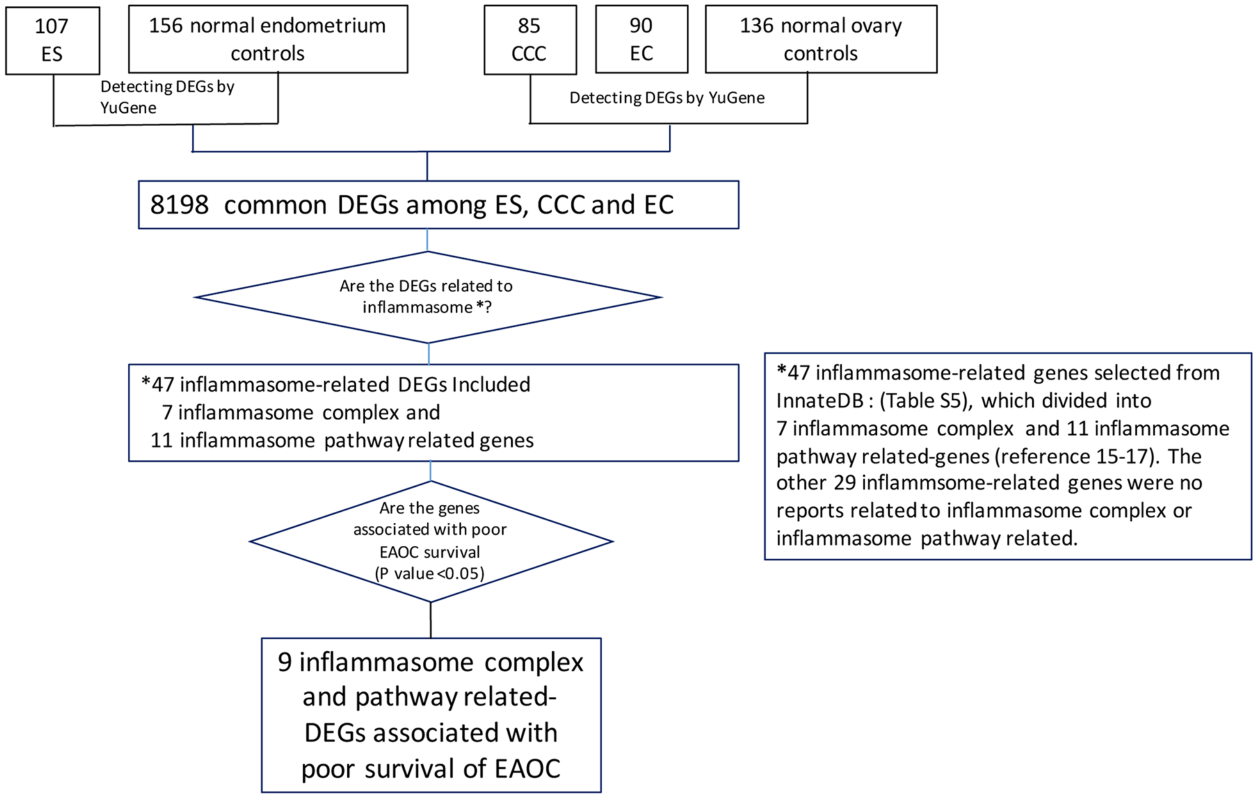


**Supplementary Figure 7: Flowchart and selection criteria of the EAOC marker genes.**
